# Effect of *Massa Medicata Fermentata* on the Gut Microbiota of Dyspepsia Mice Based on 16S rRNA Technique

**DOI:** 10.1155/2020/7643528

**Published:** 2020-09-23

**Authors:** Xiaorui Zhang, Hongling Zhang, Qinwan Huang, Jilin Sun, Renchuan Yao, Jin Wang

**Affiliations:** ^1^Chengdu University of Traditional Chinese Medicine, Chengdu, Sichuan, China; ^2^Sichuan Fuzheng Pharmaceutical Co. Ltd., Chengdu, Sichuan, China; ^3^Sichuan Fermentation Traditional Chinese Medicine Engineering Research Center, Chengdu, Sichuan, China

## Abstract

*Massa Medicata Fermentata* (MMF) is a traditional Chinese medicine (TCM) for treating indigestion and its related disorders. This study analyzes the effect of MMF on intestinal microorganisms in dyspepsia mice based on 16S rRNA technology. We take a dyspepsia model caused by a high-protein, high-calorie, high-fat diet. The 60 specific-pathogen free Kunming (SPF KM) mice were randomly divided into a model group (*n*=12), an MMF group (LSQ group, *n*=12), a Jianweixiaoshi group (JWXS group, *n*=12), a domperidone group (DP group, *n*=12), and a blank group (*n*=12). On the seventh day of administration, mice were fasted and deprived of water. After 24 h, take the second feces of stress defecation in mice under strict aseptic conditions and quickly transfer them to a sterile cryotube. This study comprehensively evaluates the *α*-diversity, *β*-diversity, flora abundance and composition of each group of miceʼs intestinal microorganisms, and their correlation with functional dyspepsia based on the 16S rRNA gene sequencing technology. After modeling, some dyspepsia reactions, proximal gastric relaxation reduction, and intestinal microflora changes were noted. Dyspepsia mice showed dyspepsia reactions and proximal gastric relaxation reduction, characterized by a significant decrease of contents of gastrin (*P* < 0.01) and cholinesterase (*P* < 0.01). MMF can improve dyspepsia symptoms and promote proximal gastric relaxation. Significant intestinal flora disorders were found in dyspepsia mice, including downregulation of *Bacteroidetes*, *Lactobacillus,* and *Prevotellaceae* and upregulation of *Proteobacteria*, *Verrucomicrobia*, *Epsilonbacteraeota*, *Firmicutes*, *Lachnospiraceae NK4A136 group*, and *Lachnospiraceae*. MMF could alleviate intestinal microflora disturbance, and the regulation effect of MMF on *Bacteroidetes*, *Verrucomicrobia*, and *Epsilonbacteraeota* was more reliable than that of Jianweixiaoshi tables and domperidone. The intestinal microflora may be correlated with the promoted digestion of MMF.

## 1. Introduction

Dyspepsia, also known as functional dyspepsia (FD), is a common functional gastrointestinal disorder (FGID), mainly characterized by postprandial fullness, early satiety, upper abdominal pain, upper abdomen burning, belching, nausea, and other dyspeptic symptoms [1]. FD incidence is high, 11%–20% in Western countries and 11.8%–23.8% in China [2, 3]. Most patients have chronic, recurrent symptoms that affect their quality of life to varying degrees. The pathogenesis has not yet been completely elucidated, and most of them are considered to be associated with abnormal gastrointestinal motility and high visceral sensitivity [4]. Small intestinal bacterial overgrowth (SIBO) can manifest various digestive and malabsorption manifestations, such as abdominal pain, bloating, diarrhea, and weight loss [5]. Gastric motility disorder can cause food siltation and bacterial growth in the upper digestive tract, leading to SIBO [6]. Therefore, gastrointestinal motility abnormalities may be related to the occurrence of SIBO in patients with FD, and its potential clinical significance is worth attention. Many FD patients have stubborn and repetitive symptoms, and their refractory features mainly include complex dysmotility and visceral hypersensitivity. In addition to the high tension of the cerebral cortex, they are also involved in intestinal microecology changes and imbalance of mediation mechanisms of the gastrointestinal mucosal immune-inflammatory response [7]. Besides, the combined use of probiotics improves dyspeptic symptoms, suggesting that intestinal flora disorders may be involved in FD's pathogenesis [8], which also provides new ideas for treating FD.

*Massa Medicata Fermentata* (MMF), also known as *Shenqu* and *Liuqu*, is a traditional Chinese medicine for treating indigestion and its related disorders. MMF's primary functions are linked to protecting the spleen and stomach and promoting digestion, which is especially suitable for children's functional dyspepsia in clinical practice. MMF comprises a certain proportion of flour, wheat bran, rice bean powder, and bitter apricot seed powder. And then it is added to water extract of *Artemisia centifolia*, *Polygonum hydropiper* L., and *Xanthium sibiricum*, to make a mixture of consistent humidity. The ratio of the above raw materials used is in turn 25 : 50 : 1:1 : 5:5 : 5. Then it is pressed into a small square with a fixed mold. It is fermented under a constant temperature of 36°C and humidity of 75% [9].

Our previous research confirmed that MMF protects the spleen and stomach and promotes digestion functions. However, we did not analyze the specific flora. Therefore, we used 16S rRNA sequencing to study the intestinal flora's corresponding changes to understand the MMF district's action mechanism.

## 2. Materials and Methods

### 2.1. Animals

A total of 30 male SPF KM mice and 30 female SPF KM mice (18–22 g) were obtained from Chengdu Dashuo Biotechnology Co., Ltd. (Chengdu, China). Depending on the Declaration of Helsinki, all animals received humane care promulgated in 1964 and amended in 1996 [10]. All experimental protocols were approved by the Animal Care and Use Committee of Chengdu Municipal Hospital of Traditional Chinese Medicine, Chengdu, China (approval no. SCXK (Chuan) 2015–30). The animals were kept in a sterile animal room, which includes individually ventilated cages, and the feed and drinking water was subjected to autoclaving during the test.

### 2.2. Liquid Medicine Preparation

#### 2.2.1. Preparation of MMF Water Extract

Take an appropriate amount of MMF sample, mix with ten times of water, soak for 30 min. Water bath refluxes extraction twice, one hour each time. Filter with 200 mesh gauze and combine both filtrates. Finally, the rotary evaporation method was utilized to concentrate to the desired concentration.

#### 2.2.2. Preparations of Jianweixiaoshi and Domperidone Suspension

Take the appropriate amount of Jianweixiaoshi tablet and domperidone tablet into a fine powder. Then add an appropriate amount of pure water to dissolve the suspension with the required concentration. It was refrigerated at 4°C. It should be heated to room temperature when using it.

### 2.3. Modeling and Treatment

After adaptation to feeding, 12 mice were randomly selected as a blank group, and the remaining mice were made into a dyspepsia model. A blank group was given a regular diet, and the other groups were fed a self-made high-protein, high-calorie diet. The diet comprises soy flour, fish, flour, milk powder in a ratio of 2 : 1:1 : 1 and thoroughly mixed with water. Then it is made into a biscuit shape and dried. At the same time, feed 50% milk 2 mL·kg^−1^ every day. They were free to eat and drink for seven days, causing the dyspepsia model. The body weight, abdominal circumference, and average food intake and feces of each mouse were measured and recorded on days 2, 5, and 8. Compared with the blank group, food intake and feces were significantly reduced, abdominal fullness and abdominal circumference increased, and the dyspepsia model was successfully simulated. After successful modeling, the 48 mice were randomly divided into a model group, an MMF group (LSQ group), a Jianweixiaoshi group (JWXS group), a domperidone group (DP group), and a blank group. Normal and model mice were given normal saline by gavage every day (50 ml·kg^−1^), the LSQ group was given MMF water extract by gavage every day (4.8 g·kg^−1^), the JWXS group was given Jianweixiaoshi suspension by gavage every day (2.88 g·kg^−1^), and the DP group was given domperidone suspension by gavage every day (0.006 g·kg^−1^), with continuous administration for 7 d.

### 2.4. Sample Collection

On the seventh day of administration, mice were fasted and deprived of water. After 24 h, take the second feces of stress defecation in mice under strict aseptic conditions and quickly transfer them to a sterile cryotube. The cryotubes were stored in a −80°C refrigerator for later use. The orbital venous plexus was used to collect blood in mice. Take a rigid glass dropper (capillary diameter of 0.5–1.0 mm) to puncture from the eye's inner corner. The inclined plane of the needle first points to the eyeball and then turns 180 degrees so that the inclined plane faces the orbit's posterior boundary. The penetration depth is about 2–3 mm. When getting the required amount of blood, we should remove the pressure on the neck. At the same time, the blood collector was pulled out to avoid bleeding from the puncture hole. If there is bleeding, use cotton ball compression hemostasis. Then apply centrifugation at 3000 rpm for 15 min, and the serum was collected and stored in the refrigerator at −70°C for later use. Follow the three kit instructions to determine the contents of gastrin, cholinesterase, and nitric oxide in serum. Finally, animals were humanely sacrificed via cervical spine dislocation.

### 2.5. Determination of Gastrin, Cholinesterase, and Nitric Oxide in Serum

Take frozen mouse serum and test gastrin, cholinesterase, and nitric oxide according to the instructions.

### 2.6. DNA Extraction and Purification

According to the manufacturer's instructions, the mice fecal bacterial genome total DNA was extracted using a soil DNA kit according to the kit standard, and the extracted genomic DNA was detected using 2% agarose gel electrophoresis and a super differential spectrophotometer (Thermo Fisher). The purified DNA extract was stored at −80°C until use.

### 2.7. MetaVxTM Library Preparation and Illumina MiSeq Sequencing

Take the mouse's feces, extract DNA, and use 0.8% agarose gel electrophoresis to detect the DNA. The purified genomic DNA was amplified by polymerase chain reaction (PCR) according to experimental instructions. 515F (5′-GTGYCAGCMGCCGCGGTAA-3′) and 806R (5′-GGACTACHVGGGTWTCTAAT-3′) were used as primers [11, 12]. Perform PCR product detection, purification, and quantification on the test sample. Use the TruSeq DNA PCR-Free Sample Preparation Kit to construct the library. After the quantified library has been quantified and the library is qualified, it is sequenced using the PE250 mode of the MiSeq 3000 platform PE300 of Chengdu Luoning Biotechnology Co., Ltd. The original offline data obtained by sequencing are spliced. Filter to obtain the high-quality target sequence required for subsequent analysis. The library was constructed using the TruSeq DNA PCR-Free Sample Prep Kit (Illumina, FC-121-3001/3003). After the constructed library was qualified and tested by the library, it was sequenced using the MiSeq 3000 platform PE300 mode sequencing, sequencing kit. Use the Hiseq Rapid SBS Kit v2 (Illumina, FC-402-4023 500 Cycle). DNA samples were quantified using a Qubit 2.0 Fluorometer (Thermo Scientific). V4 hypervariable regions of microbial 16S rDNA were selected for generating amplicons and following taxonomy analysis. Synthesis of specific primers with Barcode for the 16S rRNA gene in fecal DNA extracts PCR amplification, and the corresponding sequences of primers are 515F (5′-GTGYCAGCMGCCGCGGTAA-3′) and 806R (5′-GGACTACHVGGGTWTCTAAT-3′), respectively. Each 25 *μ*L system included 1x PCR buffer, 1.5 mM MgCl2, 0.4 *μ*M dNTPs forward and reversed primers of 1.0 *μ*M each, 0.5 U KOD-Plus-Neo enzyme (TOYOBO), and ten ng template. The PCR procedure consisted of starting at 94°C for 1 min and then 30 cycles (denaturation at 94°C for 20 s, annealing at 54°C for 30 s, and extension at 72°C for 30 s) and finally at 72°C for 5 min. Three replicates were performed for each sample. After the end of the PCR, the PCR products of all the same samples were mixed and subjected to electrophoresis detection. A recovery kit recovered the PCR products, and the target DNA fragment was eluted with TE buffer. PCR was mixed with 1/6 volume of 6 × loading buffer and detected by agarose gel electrophoresis. Take the strip for recycling and recycle the QIAquick Gel Extraction Kit (QIAGEN).

### 2.8. Data Analysis

Based on Usearch (http://drive5.com/uparse/) software, OTUSE algorithm [13] is used to perform OTU clustering at 97% consistency level, and the highest frequency sequence in each OTU is selected as the representative one. Annotated analysis was performed using the UCLUST classification [14] and the SILVA database (Rlease_123 http://www.arb-silva.de/). Representative sequences were subjected to multiple alignments using PyNAST. Use FastTree [15] to build a phylogenetic tree. Each sample was homogenized and resampled, with the least amount of data in the sample. Community composition analysis was performed using the R language [16] for various data transformations. Use the ggplot [17] package to the plot. Differential species analysis uses the *Python* LEfSe package. Random forest analysis uses the *R* language random forest package. The metastatic analysis is performed using the *R* language. Correlation analysis uses the cor.test function of Rʼs stats package. Use the *R* language psych and corrplot package to analyze the relationship between the promotion of digestion and decreased gastric relaxation and bacteria. The analysis results were statistically significant, with *P* < 0.05. Data were expressed as mean ± standard deviation (*M* ± *S*), basic statistical analysis and charting were performed using SPSS Statistics v17.0 statistical analysis software, one-way ANOVA, and LSD, and Dunnett's T3 method was used. After the two-two comparative analysis, those who do not conform to the normal distribution use the rank-sum test. *P* < 0.05 was considered statistically significant.

## 3. Results

### 3.1. The Effect on Mouse Weight, Dominant Girth, and Stool Weight

According to Table 1, there were no significant differences in weight, dominant girth, and stool weight in each group before modeling. After modeling, compared with the blank group, the model groupʼs weight and stool weight decreased, and the abdominal girth increased. After treatment, the weight, abdominal girth, and stool weight of the LSQ group, the JWXS group, and the DP group recovered nearly to the blank group. The model group was not recovered. The results showed that the method used throughout this experiment could successfully establish a dyspepsia mouse model.

### 3.2. Effects on Serum Gastrin, Cholinesterase, and Nitric Oxide

It can be seen from Table 2 that, compared with the blank group, the gastrin and cholinesterase in the model group were significantly reduced (*P* < 0.01), and nitric oxide was significantly increased (*P* < 0.01). Compared with the model group, the administration groups can significantly increase the levels of gastrin and cholinesterase (*P* < 0.01, *P* < 0.05) and can also significantly reduce the level of nitric oxide (*P* < 0.01, *P* < 0.05).

### 3.3. Analysis of the Diversity of Intestinal Flora in Mouse

**Figure 1:**
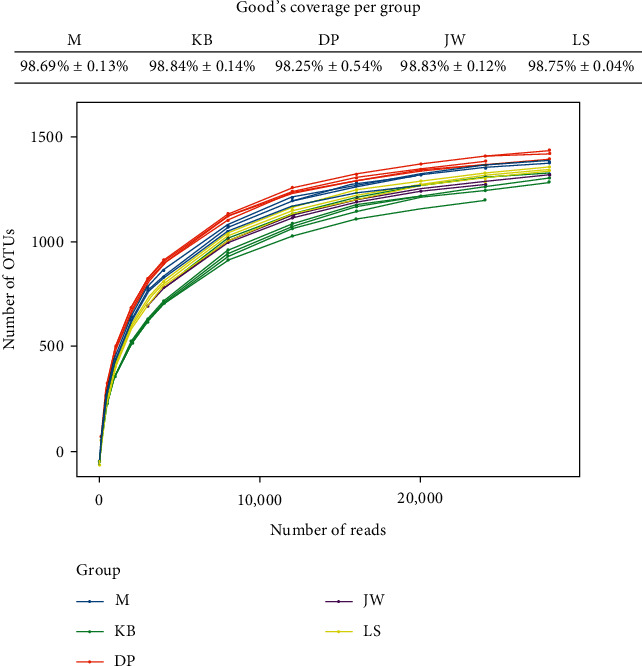
Rarefaction curve of intestinal flora in the mouse from each group.

**Figure 2:**
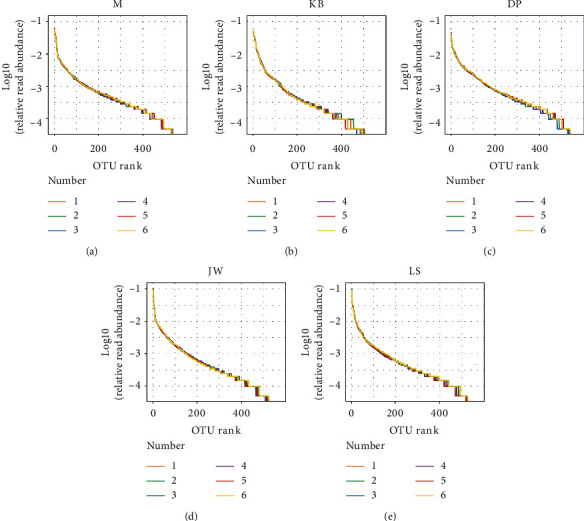
Rank-Abundance curves of intestinal flora in the mouse from each group.

We analyzed the sample dilution curve, rank-abundance curve, and alpha diversity index to study the richness, uniformity, and diversity of the mouse intestinal flora; see Table 3 and Figures 1 and 2. The large dilution curve and rank-abundance curve indicate that the sequencing depth is sufficient and can cover most species (all sample coverage is higher than 0.99). There are differences between the OTU, chao1, and Shannon indexes of the model group and the blank group (*P* < 0.01, *P* < 0.05), and there are also significant differences between the OTU, chao1, Shannon, Simpson, and PD indexes of the administration groups and the model group (*P* < 0.01, *P* < 0.05). According to the data, MMF can significantly increase the species number and diversity of intestinal flora. The reason may also be related to the fact that MMF is a fermented product. Jianweixiaoshi tablet can significantly reduce the number of intestinal flora and be close to the blank group mice.

**Figure 3:**
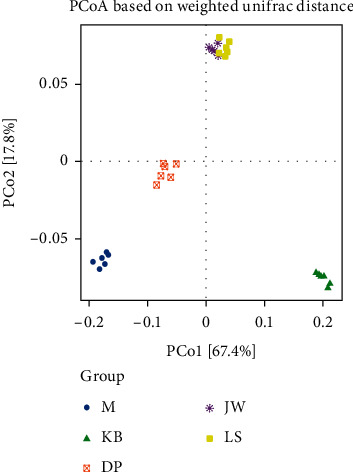
PCoA score distribution of intestinal flora in the mouse from each group.

The principal coordinate analysis (PCoA) is shown in Figure 3, and it is found that the percentages explained by PC01 and PC02 to the overall variance are 67.4% and 17.8%, respectively. The high-dose LSQ group is very close to the JWXS tablet group and completely separated from the DP group, and the blank group is wholly separated from the model group. It shows that dyspepsia mice's intestinal flora has changed significantly, and MMF, Jianweixiaoshi tables, and domperidone tend to reverse this change.

**Figure 4:**
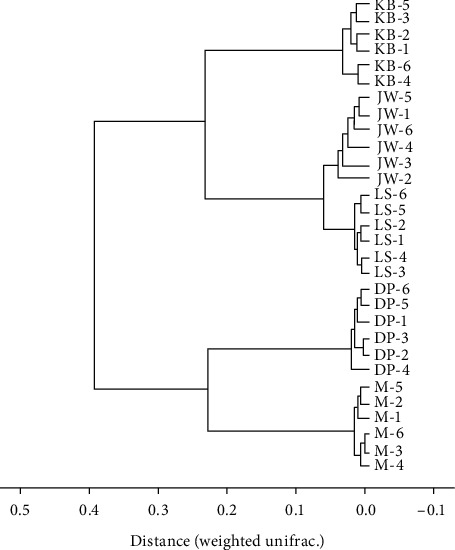
Cluster Analysis Tree of intestinal flora in the mouse from each group.

To intuitively compare the similarity between different samples, cluster analysis is used for calculation and graphing. Based on different distance matrix for cluster analysis, the results are shown in Figure 4. The closer the sample in the figure is, the shorter the branch length is, indicating that the two samples' community structure is similar. The results showed that the distance between the model group and the blank group was the longest, and the distance between the LSQ group and the blank group was between the two control groups, indicating that the internal flora structure was close to the blank group after administration. The blank group is the farthest from the model group, and the closest to the blank group are the JWXS group, the LSQ group, and the DP group.

### 3.4. Analysis of the Structural Composition of Intestinal Flora in Mice

We can understand OTU's annotation status at the phylum and genus levels in mice's intestinal flora by analyzing the microflora in the feces of accumulating mice.

**Figure 5:**
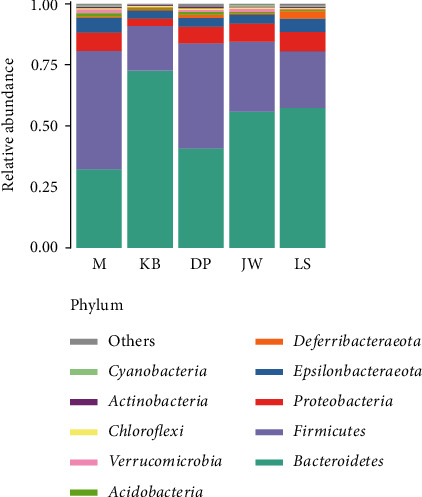
Relative abundance of community classification of intestinal flora in the mouse from each group at the phylum level.

At the door level, the five groups are mainly *Bacteroidetes* (32.03%–72.60%), *Firmicutes* (18.44%–48.82%), *Proteobacteria* (3.18%–7.86%), and *Epsilonbacteraeota* (0.67%–5.83%). Various types of bacteria and their proportion are above 94%; see Figure 5. Compared with the blank group, the proportion of *Bacteroidetes* in the intestinal flora of the food product model mice was significantly reduced (*P* < 0.01), from 72.6% to 32%, while the contents of the LSQ group, JWXS group, and DP group were 57.5%, 56.1%, and 40.7%; the proportion of *Firmicutes* increased significantly (*P* < 0.01), from 18.4%–48.8%, while the contents of LSQ group, JWXS group, and DP group were 23.2%, 28.5%, and 42.9%. All three administration groups alleviated this change to a certain extent.

**Figure 6:**
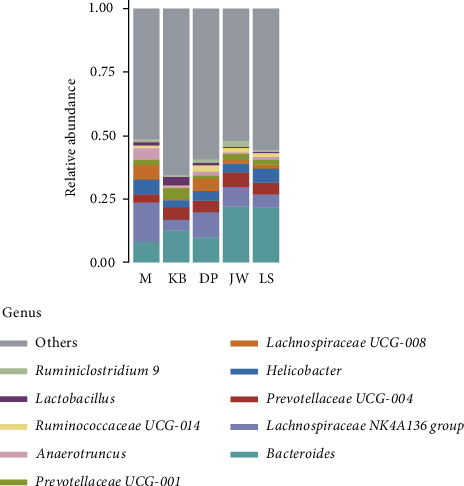
Relative abundance of community classification of intestinal flora in the mouse from each group at the genus level.

**Figure 7:**
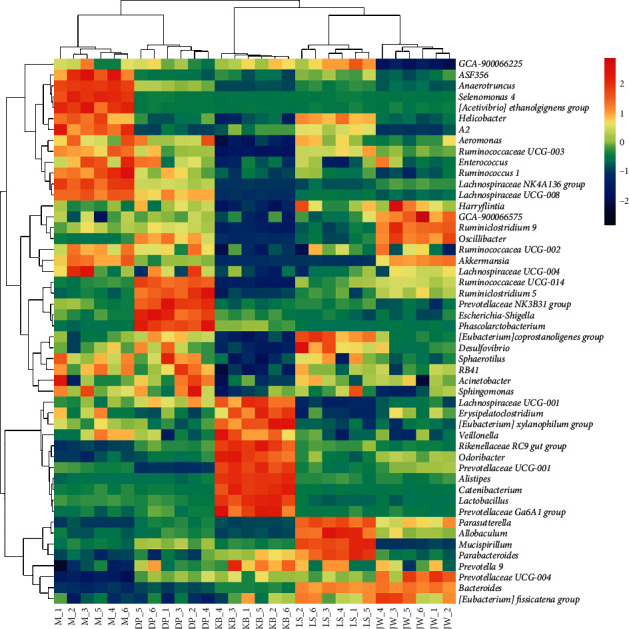
Heatmap of species with the high abundance of intestinal flora in the mouse from each group at the genus level.

At the genus level, the OTU annotations of the five groups are *Bacteroides* (8.39%–22.23%), *Lachnospiraceae NK4A136 group* (4.12%–15.48%), *Prevotellaceae UCG-004* (3.10%–5.80%), *Helicobacter* (0.67%–5.83%), *Lachnospiraceae UCG-008* (0.45%–5.90%), *Prevotellaceae UCG-001* (1.33%–4.53%), *Ruminococcaceae UCG-014* (0.56%–2.40%), *Anaerotruncus* (0.38%–4.25%), *Lactobacillus* (0.66%–3.55%), and *Parabacteroides* (0.88%–1.96%) mainly; see Figures 6 and 7. Compared with the blank group, the OTU in the model group was annotated as *Lachnospiraceae NK4A136 group*(*P* < 0.01), *Helicobacter*(*P* < 0.01), *Lachnospiraceae UCG-008*(*P* < 0.01), and *Anaerotruncus*(*P* < 0.01). *Bacteroides* decreased significantly (*P* < 0.01); *Prevotellaceae UCG-001*(*P* < 0.01) and *Lactobacillus*(*P* < 0.01) decreased significantly.

**Figure 8:**
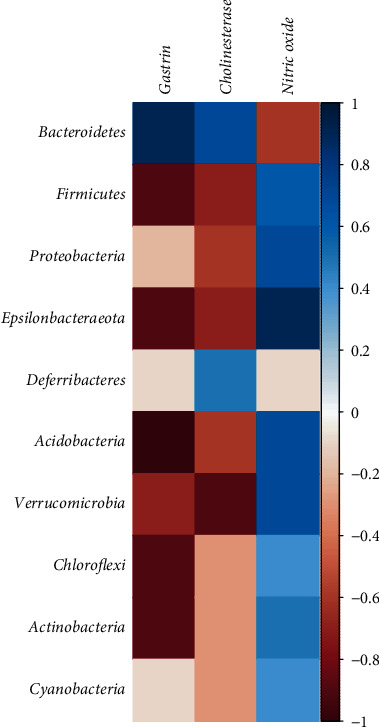
Relationship between the promotion of digestion, decreased gastric relaxation, and microbial community on intestinal flora in the mouse from each group at the phylum level.

The correlation between 10 high-abundance phyla and the promotion of digestion and decreased gastric relaxation is shown in Figure 8. It was found that, in terms of promoting digestion, gastrin expression is highly correlated with *Bacteroidetes*. In terms of decreased gastric relaxation, the expression of cholinesterase has a high negative correlation with *Verrucomicrobia*.

## 4. Discussion

Human gut microbes are the “second genome” of the human body [18–20]. Changes in nutrient utilization and synthesis may accompany changes in the intestinal microbiota in species richness, diversity, composition, and function. These changes will have a profound impact on the host's physiological response [21]. Many studies have shown that FD and digestive system dysfunction are closely related [22]. MMF and many microorganisms, such as yeast and mold produced in the fermentation process, can improve intestinal microflora disorder symptoms in mice. It can regulate and protect the digestive system of animals and improve the intestinal flora [23].

Studies have shown that gut microbiota is closely related to indigestion [24]. FD patients will have substantial fullness symptoms after a meal due to the stomach's delayed emptying. Simultaneously, many findings using a gastric barostat have shown reduced proximal gastric relaxation in response to a meal in FD patients [25, 26]. Insufficient accommodation of the proximal stomach during and after a meal's ingestion may be accompanied by increased intragastric pressure and activation of mechanoreceptors in the gastric wall, thus inducing symptoms [27]. MMF's pharmacological effect is mainly manifested in promoting food hydrolysis and improving gastrointestinal motility to enhance digestion [28]. Studies have shown that MMF can promote the movement of mouse ileal smooth muscle [29]. This study found that MMF, Jianweixiaoshi, and domperidone can alleviate abdominal fullness symptoms in mice, and these changes may be related to the intestinal flora. Through the analysis of the relationship between the promotion of digestion, decreased gastric relaxation, and microbial community, it is found that MMF ability to improve intestinal ecology and promoting gastric emptying may have a specific correlation with intestinal flora and *Bacteroidetes*, but this needs to be confirmed by further experimental research.

Standard or “healthy” gut microbiota mainly includes *Firmicutes* (about 50%–75%) and *Bacteroidetes* (about 10%–50%) [18]. The *Bacteroidetes* of the dyspepsia mice was significantly reduced, which is consistent with the research results of Zhuang et al. [30], and MMF could reverse this change. This study indicates that the increase in intestinal microflora diversity in dyspepsia mice may be related to disturbance of intestinal microbiota and the increase of pathogenic bacteria (such as *Helicobacter*). Intestinal flora disorder inhibits short-chain fatty acid production and conversion (SCFA), one of the factors that induce FD. The most crucial SCFA in the intestine is acetic acid, propionic acid, and butyric acid [31]. Short-chain fatty acids are also the main products of protein degradation and amino acid fermentation [32]. Propionic acid-producing bacteria mainly belong to *Bacteroidetes*, and MMF can significantly increase the expression of *Bacteroides*. At the same time, this study found a negative correlation between *Verrucomicrobia* and cholinesterase. The muscle can relax afterward, rather than remain locked in a tense state; the acetylcholine must be broken down by a cholinesterase. The *Verrucomicrobia* of the dyspepsia mice is significantly increased, and MMF can reverse this change. MMF may restore the cholinesterase of dyspepsia mice by inhibiting the number of *Verrucomicrobia*, thereby obtaining proximal gastric relaxation and improving dyspepsia symptoms.

Besides, studies have shown that MMF contains artemisinin, rutin, oleanolic acid, amygdalin, and quercetin, which have an excellent inhibitory effect on common intestinal pathogens, and it also has a good repair effect on the wounds on the surface of the digestive tract [33, 34]. It shows that traditional Chinese medicine MMF can increase the number of beneficial bacteria in the intestine, reduce the number of aerobic bacteria, and have a therapeutic effect on dyspepsia.

In summary, MMF can play a therapeutic role by regulating intestinal flora disturbance in dyspepsia mice.

## 5. Conclusion

In this study, the 16S rRNA technique was used to provide an unexpected and unbiased analysis of MMF's action mechanism. MMF is a fermented medicine commonly used in Chinese medicine. First, assessing the changes in serum metabolites, we found that MMF has a specific effect on digestive function. Using high-throughput sequencing technology to detect intestinal microbes, we found that MMF plays a functional role in regulating intestinal microbes. It is consistent with traditional Chinese medicine theory: “MMF protects the spleen and stomach.” By analyzing the correlation between 10 high-abundance bacteria and related factors of the digestive system, we further found that MMF plays a role in protecting the spleen and stomach and promoting digestion by regulating intestinal expression microorganisms such as *Bacteroidetes* and *Verrucomicrobia*.

The research provides a valuable reference model for explaining Chinese medicine theory, expands MMF's potential applications, and provides reasonable goals and directions for further mechanism research.

## Figures and Tables

**Table 1:** Comparison of mouse weight, abdominal girth, and stool weight $\left(\bar{X}\pm ,\;n=12\right)$.

Groups	Weight (g)			Abdominal girth (cm)			Stool weight (g·g^−1^)		
	Before modeling	After modeling	After administration	Before modeling	After modeling	After administration	Before modeling	After modeling	After administration
Blank group	24.68 ± 0.90	30.46 ± 1.24	32.61 ± 1.55	8.07 ± 0.18	8.44 ± 0.18	8.94 ± 0.27	0.09	0.12	0.12
Model group	24.56 ± 0.98	28.02 ± 1.03^*∗∗*^	29.25 ± 1.35^*∗∗*^	8.02 ± 0.18	9.08 ± 0.19^*∗∗*^	9.37 ± 0.21^*∗∗*^	0.09	0.07	0.08
LSQ group	24.65 ± 1.14	28.31 ± 0.69	30.54 ± 1.64^#^	8.10 ± 0.16	9.21 ± 0.23	9.12 ± 0.29^#^	0.09	0.07	0.10
JWXS group	24.59 ± 0.73	28.52 ± 1.38	31.46 ± 1.65^##^	8.12 ± 0.19	9.19 ± 0.24	9.08 ± 0.29^#^	0.09	0.07	0.10
DP group	25.21 ± 1.24	28.65 ± 1.43	30.86 ± 2.01^#^	8.12 ± 0.11	9.17 ± 0.22	9.02 ± 0.26^##^	0.09	0.07	0.10

Compared with the blank group: ^*∗*^*P* < 0.05; ^*∗∗*^*P* < 0.01. Compared with the model group: ^#^*P* < 0.05; ^##^*P* < 0.01.

**Table 2:** Content of three hormones in mouse serum $\left(\bar{X}\pm s,\;n=12\right)$.

Groups	Dose/(g·kg^−1^)	Gastrin (*μ*g/mL)	Cholinesterase (nmol/l)	Nitric oxide (*μ*mol/l)
Blank group	—	2.54 ± 0.31	152.69 ± 4.08	57.75 ± 4.02
Model group	—	1.85 ± 0.18^*∗∗*^	132.24 ± 5.20^*∗∗*^	88.12 ± 4.57^*∗∗*^
LSQ group	4.8	2.40 ± 0.29^##^	145.55 ± 2.82^#^	77.67 ± 3.97^#^
JWXS group	2.88	2.45 ± 0.35^##^	144.54 ± 3.14^#^	76.61 ± 3.36^#^
DP group	0.006	2.23 ± 0.28^#^	148.05 ± 2.76^##^	71.20 ± 2.78^##^

Compare with the blank group: ^*∗*^*P* < 0.05; ^*∗∗*^*P* < 0.01. Compare with the model group: ^#^*P* < 0.05; ^##^*P* < 0.01.

**Table 3:** Comparative analysis of the indicators of alpha diversity analysis in two groups $\left(\bar{X}\pm s\right)$.

Groups	OTU	Chao1	Shannon	Simpson	*P*D
Blank group	852.17 ± 30.14	1048.24 ± 76.17	4.79 ± 0.03	0.98 ± 0.0006	67.7 ± 1.87
Model group	993.83 ± 26.93^*∗*^	1220.22 ± 71.84^*∗*^	5.02 ± 0.02^*∗∗*^	0.98 ± 0.0004	75.65 ± 2.35
LSQ group	1187.17 ± 193.58^##^	1502.37 ± 305.20^##^	5.44 ± 0.09^##^	0.99 ± 0.0005^##^	89.37 ± 15.22^##^
JWXS group	886.83 ± 30.69^#^	1083.76 ± 62.52	4.91 ± 0.02^##^	0.98 ± 0.001	69.38 ± 2.24
DP group	926.83 ± 23.42	1138.82 ± 29.58	4.91 ± 0.03^##^	0.98 ± 0.0008	72.36 ± 2.29

Compared with the blank group: ^*∗*^*P* < 0.05; ^*∗∗*^*P* < 0.01. Compared with the model group: ^#^*P* < 0.05; ^##^*P* < 0.01.

## Data Availability

The datasets used and analyzed during the current study are available from the corresponding author on reasonable request.
